# “It’s like heaven over there”: medicine as discipline and the production of the carceral body

**DOI:** 10.1186/s40352-020-00107-5

**Published:** 2020-02-08

**Authors:** Jason E. Glenn, Alina M. Bennett, Rebecca J. Hester, Nadeem N. Tajuddin, Ahmar Hashmi

**Affiliations:** 1 Department of History and Philosophy of Medicine, University of Kansas Medical Center, Kansas City, KS 66160 USA; 2 Regional Ethicist, Kaiser Permanente, Northern California, Oakland, CA 94612 USA; 3 Department of Science, Technology and Society, Virginia Polytechnic Institute and State University, Blacksburg, VA 24061 USA; 4 Department of Internal Medicine, Baylor College of Medicine, Houston, TX 77030 USA; 5 Department of Family Medicine, Faculty of Medicine, Chiang Mai University, Chiang Mai, 50220 Thailand; 6 Shoklo Malaria Research Unit, Mahidol-Oxford Research Unit, Faculty of Tropical Medicine, Mahidol University, Mae Sot, 63110 Thailand

**Keywords:** Correctional managed care, Incarcerated patients, Prisoners, Academic medical centers, Incarceration, Medical students, Medical residents, Critical prison studies

## Abstract

**Background:**

Correctional systems in several U.S. states have entered into partnerships with Academic Medical Centers (AMCs) to provide healthcare for people who are incarcerated. This project was initiated to better understand medical trainee perspectives on training and providing healthcare services to prison populations at one AMC specializing in the care of incarcerated patients: The University of Texas Medical Branch at Galveston (UTMB). We set out to characterize the attitudes and perceptions of medical trainees from the start of their training until the final year of Internal Medicine residency. Our goal was to analyze medical trainee perspectives on caring for incarcerated patients and to determine what specialized education and training is needed, if any, for the provision of ethical and appropriate healthcare to incarcerated patients.

**Results:**

We found that medical trainees grapple with being beneficiaries of a state and institutional power structure that exploits the neglected health of incarcerated patients for the benefit of medical education and research. The benefits include the training opportunities afforded by the advanced pathologies suffered by persons who are incarcerated, an institutional culture that generally allowed students more freedom to practice their skills on incarcerated patients as compared to free-world patients, and an easy compliance of incarcerated patients likely conditioned by their neglect. Most trainees failed to recognize the extreme power differential between provider and patient that facilitates such freedom.

**Conclusions:**

Using a critical prison studies/Foucauldian theoretical framework, we identified how the provision/withholding of healthcare to and from persons who are incarcerated plays a major role in disciplining incarcerated bodies into becoming compliant medical patients and research subjects, complacent with and even grateful for delayed care, delivered sometimes below the standard best practices. Specialized vulnerable-population training is sorely needed for both medical trainees and attending physicians in order to not further contribute to this exploitation of incarcerated patients.

## Introduction

“As a result of this new restraint, a whole army of technicians took over from the executioner, the immediate anatomist of pain: warders, doctors, chaplains, psychiatrists, psychologists, educationalists; by their very presence near the prisoner, they sing the praises that the law needs: they reassure it that the body and pain are not the ultimate objects of its punitive action. Today a doctor must watch over those condemned to death, right up to the last moment—thus juxtaposing himself as the agent of welfare, as the alleviator of pain, with the official whose task it is to end life. This is worth thinking about. When the moment of execution approaches, the patients are injected with tranquillizers. A utopia of judicial reticence: take away life, but prevent the patient from feeling it; deprive the prisoner of all rights, but do not inflict pain; impose penalties free of all pain. Recourse to psycho-pharmacology and to various physiological ‘disconnectors’, even if it is temporary, is a logical consequence of this ‘non-corporal’ penality.”

– Michel Foucault, *Discipline and Punish*

Currently, there are a handful of different models for delivering secondary and tertiary healthcare to incarcerated populations (US Department of Justice, National Institute of Corrections, 2001), with most correctional healthcare delivered via contracts with publicly traded and private, for-profit corporations. Contracts with Academic Medical Centers (AMCs) represent the second most common outsourced system through which incarcerated persons receive healthcare. Correctional systems in several U.S. states, including Connecticut, Georgia, Massachusetts, New Hampshire, New Jersey, and Texas, have entered into partnerships with AMCs to provide healthcare for their incarcerated populations. In a 2015 commentary for *Academic Medicine*, Trestman et al. stress the benefits for such collaborative partnerships, including: improving public and population health, addressing the most acute and extreme health inequities, training opportunities for undergraduate and graduate medical education, decreasing risk of litigation, and “the viability of correctional health research and extramural research funding” (Trestman, Ferguson, & Dickert, 2015). In the same year, a newsletter by the Association of American Medical Colleges (AAMC) expressed similar enthusiasm and espoused similar benefits (Pelletier, 2015). These commentaries outline the benefits of correctional health for AMCs and improved outcomes over for-profit correctional health corporations, reference special training for security and boundary issues, and mention unique competencies required for delivery of correctional healthcare. However, absent from both articles is any mention of unique specialty training or considerations for ethically handling the power imbalance and vulnerability to exploitation faced by incarcerated persons who have no choice in their healthcare provider and little personal autonomy. This paper utilizes a discursive analysis to assess the perspectives of a subset of health workers who provide care to patients who are incarcerated: medical trainees. We contextualize these data against the backdrop of larger historical and structural factors that have and continue to influence the health of people who are incarcerated before advancing a vision for medical education that undermines the perpetuation of these patterns within such training environments.

Considering the special vulnerabilities of persons who are incarcerated is fundamental for practitioners providing correctional care. Incarcerated persons are often the victims of extreme poverty, trauma and abuse prior to their imprisonment (Gold, Sullivan, & Lewis, 2011; Martin, Eljdupovic, McKenzie, & Colman, 2015; Stensrud, Gilbride, & Bruinekool, 2018). Once behind bars, the prison itself becomes the most immediate structural determinant of an incarcerated patient’s health. This is reflected in the immediacy of health problems that are seen among incarcerated people, and the social inequities they reflect. Prisons maintain a strict, dehumanizing power hierarchy that is violently enforced. They have high rates of chronic infectious diseases, including tuberculosis, HIV and Hepatitis B and C viruses (Bick, 2007); and expose persons who are incarcerated to high rates of trauma-causing violence, including repeated sexual assaults, overcrowded living conditions, lack of temperature control and poor ventilation systems (Awofeso, 2010); poor sanitation and a lack of healthy food and exercise options (Baillargeon et al., 2004; Baillargeon, Black, Pulvino, & Dunn, 2000). There are also often hard-set institutional rules against implementing evidence-based public health measures that could lessen or prevent such risks, such as failing to provide opt-out HIV testing or barring the distribution of condoms (Mutter, Grimes, & Labarthe, 1994; Rubin, 2016). Many illnesses stem from conditions that predate the patient’s incarceration, only to be exacerbated by imprisonment.

Therefore, effectively caring for incarcerated patients requires understanding how they are made vulnerable by larger, structural determinants of their health. The high school completion rate for incarcerated persons is low—between 20% and 30% (Harlow, 2003; Western & Pettit, 2010). These low levels of education are associated with lower socioeconomic status, poorer access to healthcare, and higher prevalence of high risk behaviors (Baillargeon et al., 2004). Compounding this, more than half of all people in prison and jail have a mental health problem, which includes 56% of people in state prison, 45% of people in federal prison, and 64% of people in jail. Incarcerated women have much higher rates of mental health problems than men: 73% of females in state prisons, 61% in federal prisons, and 75% of women in jails (Baillargeon, Binswanger, Penn, Williams, & Murray, 2009; Prins, 2014; US Department of Justice, Bureau of Justice Statistics, 2006). Also, more than two thirds of people in jails (Karberg & James, 2005) and more than half of all people in prison have a substance use disorder or were arrested on a substance use-related offense (Fazel, Yoon, & Hayes, 2017; National Institute of Drug Abuse, 2010).

The mass incarceration of mentally ill persons with co-morbid substance use disorders is a prime example of larger structural determinants impacting the health of incarcerated persons. The closing of mental health hospitals that began in the 1960s (Primeau, Bowers, Harrison, & XuXu, 2013) led to large increases of homelessness and self-medicating among those without mental healthcare coverage (Lamb & Weinberger, 2005). This was combined with a cultural shift to “broken windows” policing that more heavily criminalizes low levels of misbehavior (Garland, 2001) and targets the poor (Wacquant, 2009). When compared to mental hospitals, prisons now house ten times more persons with a mental health disorder (Haney, 2017; Torrey et al., 2014). The high rate of comorbidity suggests that over half the people incarcerated in the U.S. are there due to lack of mental healthcare and/or substance abuse treatment.

Although the commentaries published in *Academic Medicine* suggest that AMCs may help to address these health inequities, a historical perspective helps explain the nature and context within which these disparities arise, as well as the more exploitative side of what otherwise appears to be a positive partnership. Persons who are incarcerated are the most exploited population in the history of allopathic medicine, from their near exclusive use to inform 16th - eighteenth century research and teaching of anatomical form and function (Sawday, 1995), their use throughout the nineteenth century to provide clinical teaching material for medical schools (Savitt, 2007), to their systemic use for most research during the twentieth century until implementation of protections for human research subjects under the Common Rule in the Code of Federal Regulations in 1981 (Glenn, 2015; Hornblum, 1998). Indeed, the corpus of biomedical knowledge has been built upon the exploitation of people who are incarcerated (Goodman, McElligot, & Marks, 2003; Lederer, 1995; Sawday, 1995; Washington, 2006). In light of this vast history of medical exploitation of incarcerated persons for medical education and research, AMCs entering into partnerships with prison systems have a special responsibility not to replicate the exploitative abuses of the past. If incarcerated persons are now to entrust their care to this same medical establishment, special vulnerable populations training should be implemented to protect them.

The University of Texas Medical Branch (UTMB) is considered a national leader in correctional health as the only AMC to have a free-standing hospital dedicated to the specialty and tertiary care of Texas Department of Corrections (TDC) incarcerated patients. The TDC hospital is nestled among the other buildings that compose the UTMB medical center and is connected to the main university hospital via a bridge. Medical students and residents routinely treat patients in the TDC hospital, supervised by attending physicians, as part of their training and education. Given the complexities of correctional care provided above, the authors found that many learners were struggling with complex provider-patient dynamics related to the vulnerability of incarcerated persons and the greater power it afforded trainees over them, with no curricula or specialized training to help intellectually process, reflect upon, or navigate such experiences. Therefore, this study was initiated to more fully understand trainee perspectives on training and providing healthcare services to incarcerated patients. We set out to characterize perceptions and experiences of a broad spectrum of medical learners including those at the start of their training up to those in their final year of Internal Medicine residency.

### Setting: correctional care at the University of Texas medical branch

With roughly 150,000 people incarcerated in over 50 state prisons, Texas is the highest per capita incarcerator in the U.S. In addition to providing healthcare to persons in state prisons, UTMB also provides health services to those housed in city and county jails, youth detention facilities, and federal prisons, making UTMB the largest healthcare provider to incarcerated patients in the U.S. (Raimer & Stobo, 2004). It is worth noting that the legal mandate behind correctional healthcare stems from two court cases that originated in Texas due to the particularly horrid neglect and indifference to which persons incarcerated in were historically subjected. In Estelle v. Gamble (1976) the U.S. Supreme court ruled against the then director of the TDCJ, William J. Estelle, and held that all persons who are incarcerated have the right to adequate medical care while incarcerated. The court further ruled that evidence of prison officials’ “deliberate indifference” to an incarcerated person’s serious medical needs constitutes a violation of the cruel and unusual punishment clause of the 8th Amendment.

The case stemmed from an injury received by a prisoner named J.W. Gamble, who had been assigned to unload cotton bales from a truck. [Prisons in Texas and in other southern states have traditionally had an agricultural work requirement that often involved picking and baling cotton, a demonstration of the close historical relationship between prisons in the U.S. and slavery (Adamson, 1999; Childs, 2015)]. Gamble was crushed by a falling bale but denied medical treatment for his severe back pain after the accident. Suspected of malingering, he was put in solitary confinement as punishment for not working.

A court ordered right to healthcare does not include, however, yearly physicals and well-women’s exams by a general practitioner, or any other form of preventive medicine. It consists of an initial intake screening—that may not adequately assess pre-existing mental (Adams & Ferrandino, 2008) and physical health conditions—and making urgent care services available for the treatment of any emergent illnesses or injuries that arise during incarceration. Finally, there is an infirmary to administer medications, typically only once per day. Patients who are treated often suffer a systemic lack of medication continuity (Reingle Gonzalez & Connell, 2014). Previously existing chronic illnesses (such as diabetes or hypertension) are only treated sporadically and often with prior generation pharmaceuticals that are no longer the standard of care (Wilper et al., 2009).

If an incarcerated person desires some sort of primary care with regular check-ups, it requires the purchase of a health plan or requires co-payments, the cost of which far exceeds an incarcerated person’s earning potential. Thirty-nine states have authorized the collection of fees from people who are incarcerated for medical services they receive while in state prisons or county jails (Ollove, 2015). In Texas, the copay is an annual $100, assessed only if a non-emergency healthcare visit is requested (Texas Department of Criminal Justice, 2019). Persons who are incarcerated have an extremely limited earning potential, even when employed behind bars. The national average hourly wage is between $0.14 - $0.63 (Sawyer, 2017). For the average incarcerated patient then, a $100 copay is the equivalent of between 158 and 714 h of labor. Therefore, to afford a health plan or the copay fees, it usually requires someone from the outside to transfer money into the person’s commissary account. This is the same limited pot of money on which incarcerated persons depend to buy basic health hygiene items like soap and toothpaste. As-needed emergent care is not an appropriate delivery system for populations plagued by high rates of chronic illnesses or for those at high risk for diseases requiring screening for early diagnosis and prevention (Thorburn, 1995). Such a system, which so drastically dis-incentivizes preventive and routine primary care, means that serious chronic illnesses are, on average, detected at a much later stage than insured, free-world patients. Such illnesses are addressed only once the symptoms have a clearly visible outward manifestation that looks severe enough to be taken seriously by a guard and referred to medical staff (Lindquist & Lindquist, 1999). Only then does interaction with UTMB medical personnel begin.

## Methods

We conducted focus group discussions (FGD) with medical students and residents in the Internal Medicine (IM) Residency Program at UTMB, between December 2014 and February 2015. Researchers worked with course coordinators to identify potential medical trainees from UTMB’s School of Medicine and the IM Residency Program. We chose only IM residents as opposed to other residency programs at UTMB as IM residents are the trainees who most frequently care for incarcerated patients. Aside from a group of first-year medical students who were selected at random, third- and fourth-year medical students and IM residents were selected at random from trainees who had completed a rotation in the TDC Hospital (Table 1).

**Table 1:** Participant demographics (MS: medical student; PGY: post-graduate year, Internal Medicine residents)

Medical Trainee Level	Women	Men	Total
MSI	7	3	10
MSII	0	0	0
MSIII	3	2	5
MSIV	1	1	2
PGY-1	3	0	3
PGY-2	4	2	6
PGY-3	0	4	4
Total (*n.*)	18	12	30

The FGD guide was drawn from the Attitudes Toward Prisoners scale (Melvin, Gramling, & Gardner, 1985), published studies of medical learners working with incarcerated persons and other vulnerable populations, and the experiences of AB, AH, and NT, who had performed clinical duties within the TDC Hospital as health professional trainees. The guide was designed to elicit perspectives about 1) preconceptions and anxieties trainees had before rotating in the TDC hospital; 2) their impressions on providing care to incarcerated patients; 3) ethical challenges they encountered in providing healthcare to incarcerated patients; and 4) whether the training and/or orientation they received prepared them for those challenges.

FGD were conducted on UTMB’s campus, lasted approximately 1 hour, in groups between 5 and 8 participants in size, with each FGD including participants from the same level of training. Additional FGD were conducted until the authors felt that saturation had been reached. After consenting, audio recording was initiated and participants provided their age and gender. No other identifying information was collected. FGD were transcribed verbatim. For better understanding of the transcribed interviews in relation to their contexts, observational field notes were made during and immediately after FGD about contextual characteristics, atmosphere and relevant non-verbal communications. Recordings, transcripts and field notes were password-protected and kept on a password-secure computer.

### Data analysis

Co-authors AB, AH, and JG performed line-by-line inductive analysis of the transcripts, using open coding. Following a grounded theory approach, codebooks from three investigators were compiled separately, then repeated discussions were held to finalize a codebook with agreed upon definitions, and then transcripts were re-analyzed. Inter-reliability analysis was performed using this master file to identify discrepant interpretations requiring further discussion. Individual investigators performed thematic analysis as a final phase, identifying emergent themes, which were then discussed and compiled.

In addition to the emergence of the above-mentioned meta-themes, we found the dataset well-suited to a critical prison studies analysis relying heavily on Michel Foucault. Such an analysis interrogates the knowledge systems that make particular social arrangements and power hierarchies thinkable. In supplying healthcare to prisoners for the dual purpose of teaching and knowledge acquisition, we see Foucault’s concept of biopower illuminated starkly in the interactions between incarcerated patients and medical trainees. Foucault defined biopower as “an explosion of numerous and diverse techniques for achieving the subjugations of bodies and the control of populations” (Foucault, 1976). These new techniques of subjugation were achieved, Foucault argues, by redefining what it means to be human in biological, rather than spiritual terms—a shift that occurred in the early nineteenth century. Allopathic medicine and the biomedical sciences, elaborating upon this biological conception of humanity and practicing a new mode of perception that differentiates “normal” biological function from the “pathological,” supplies the knowledge base that disciplines bodies into this new mode of being.

The early nineteenth century also sees the hospital, asylum and the prison disaggregated out of the mad houses of the eighteenth century, where paupers, criminals, the mentally ill, and the terminally sick were all thrown together and chained to the walls. For Foucault, the birth of the prison represents a new form of discipline, eschewing the use of corporal punishment to compel the body and instead targeting the psyche as a way of compelling the soul (Foucault, 1995). Yet even prior to this transformation, when disciplinary practices ran the gamut from drawing and quartering to flaying alive, there, next to the executioner’s scaffolding, stood the doctor, waiting patiently to collect what remained of the prisoner for the anatomy theatre (Sawday, 1995). Though corporal discipline remains very much in use in contemporary correctional settings, our analysis illuminates how the current system of correctional managed healthcare delivery actually achieves the kind of non-corporal discipline of which Foucault theorized.

Through this analytical lens, we were able to trace how the strained and delayed delivery of healthcare to persons who are incarcerated and the dual use of incarcerated patients as teaching material play a central role in disciplining the incarcerated body into that of the happily compliant patient and research subject.

## Results

### Malingering

Defined as “the purposeful production of falsely or grossly exaggerated physical or psychological complaints and/or symptoms with the goal of receiving a reward” (American Psychiatric Association, 2010), participants described the myriad of ways in which *malingering* is the default suspicion held by providers about every person incarcerated. Prominent within the literature on correctional medicine and within corrections blogs and discussion boards, corrections officers assume people who are incarcerated are malingering for the following reasons: to avoid criminal responsibility, reduce or alter sentencing, obtain benefits (such as Supplemental Security Income) upon release, transfer to a better location (hospital, infirmary, mental-health unit), receive lighter work duty, obtain contraband for the underground prison economy (narcotics, psychotropics), or to obtain other perks (better shoes, lower bunk, etc.) (Schoenly, 2010, 2018).

We found these assumptions mimicked by medical trainees. “Sometimes they believe this is a hotel for them and they’re faking their symptoms,” one fourth-year medical student observed. A third-year resident confirmed, stating “sometimes they fake something just to be here in the air conditioning because the units are pretty hot in the summer. And sometimes small things like watching a football game is [sic] a big deal for them.” “Chest pain,” another third-year resident chimed in, “There’s a super bowl, big game coming on, ‘I’m going to have chest pain.’” “Seizures are another big one,” a second-year resident informed us, with agreement from all the other residents interviewed in that group.

“You have some of these guys who are career criminals and they’re expert manipulators and they know what symptoms to complain about. You’ll see an influx of patients during sporting events or holidays. It’s an unfortunate truth but it’s there and sometimes those bad apples spoil the bunch and make you a little biased when you approach patients around that time,” another second-year resident complained. “It’s hard to stay unbiased when you have someone complaining of these nonspecific symptoms and you’re trying to help them but also in the back of your mind you’re thinking, ‘is there a secondary gain to why you’re here and not in your unit right now?’”

The perception of malingering persisted even after a patient was treated at the TDC Hospital. Incarcerated patients are often assumed to be lying when they complain of continued pain and suffering after their primary health complaint has been addressed. “I think that there’s a certain sense of, ‘Don’t tell the patient when they’re going to leave. You don’t want them to hold up discharge,’” one first-year resident observed. “It seems that people have an understanding—if certain patients are close to discharge, they will make up reasons to stay.” Another first-year resident affirmed this perception, stating that even though “we see malingering patients in the free-world too… there’s a little bit higher of a suspicion of malingering” in incarcerated patients.

Oftentimes, the suspicion of malingering is imparted to trainees by senior staff, creating an uncomfortable situation for learners. “We had attendings that [sic] felt that way,” one third-year medical student told us. She continued, “When patients would say they’re in pain like, [our attending would say] ‘Oh, well, they’re a criminal, they did something to get here. They’re a professional liar’ and stuff like that. So they didn’t believe they were really having pain. And sometimes they might be right but I know they wouldn’t do that with a free-world patient. Because a free-world patient could be a criminal, they could have gotten out of jail last week and you’re not going to ask every patient about that, about their criminal history before you give them pain medication. So it’s kind of hard for us to handle it whenever our attendings already don’t like them.”

Another third-year student concurred: “I want to be nice to them and usually [our attendings] just say we’re being naive or whatever. Which may be true, I don’t know.” With the unequal power dynamic that exists between learners and attendings, witnessing such discrepancies in behavior was highly discomforting. “Well it’s awkward because you don’t want to call them out on it,” a third-year medical student confided, “but, they feel that because it’s a TDC patient they have the right to judge the person’s whole life but you wouldn’t do that in the free-world. Because my service had both free-world and TDC so it was really easy to see how they treated both [types of] patients. And a lot of times it was really different.”

A few trainees noticed how many medical encounters with incarcerated patients begin on their home units with the suspicion that their illness symptoms are being faked. “[Y]ou see really neglected conditions... in TDC,” one third-year medical student told us, “because I think a lot of times when they go to their unit doctor, the doctors dismiss them. By the time they actually get brought to the hospital it’s really shocking.” A third-year resident had the same observation, stating that “because they are prisoners sometimes [the guards] think like they’re faking. That is a main factor—ignorance. Everybody puts the same label on them that they’re faking and in reality some of them really pay for it.”

### Advanced pathology

The “shocking” conditions of which the above third-year student spoke is the advanced pathology of incarcerated patients. By far, it was the most common theme independently raised by study participants, emerging in every FGD. For many trainees, any initial nervousness they held about treating incarcerated patients was quickly overshadowed by a macabre appreciation of the educational opportunity such pathology presents. “No one is telling you what the day-by-day is like so the only thing you have is what you previously came in with, for those of us with no experience of that whatsoever it was just a little scary,” a first-year medical student admitted. “Then you get here and realize, ‘Wow, this is a huge learning opportunity.’”

“I was actually looking forward to it because there’s a lot of really interesting pathology in TDC that you don’t see in the free-world. There are a lot of interesting diseases and things you don’t get to see in a developed country and that you would see in a prison population… I was really looking forward to seeing the patients there,” a fourth-year medical student confided. “You get a lot more advanced cancers especially,” a third-year medical student added, “multiple people under precautions for TB and things like that.”

Some trainees maintained stereotypical views of personal irresponsibility as the reason behind the advanced pathology they saw in incarcerated patients: “These are the people who have led riskier lives so higher risk behavior leads to more [pathology],” one first-year resident remarked. “I think a lot of them have drug addiction problems and they’re doing crazy things all the time so they end up getting [sick]. Health is not a priority, eating well and exercising, things like that, they’re not likely to do. That’s just how I see it.”

A few trainees made the connection between advanced pathology and institutional neglect. “When they’re at their unit, a lot of times the unit doctor may neglect them until they’re really, really sick. By the time they get here they can be really bad off and you see that pathology,” a third-year resident told us. “[T]he majority of what I’ve seen of the incarcerated patient population doesn’t even make it to you in time,” a first-year student confirmed. “You’re not dealing with the buildup of the disease, they’re coming to you and they’re already with a disease fully developed.”

“It’s access while they’re in the prison system,” a second-year resident asserted. “We see a lot of things, we see really sick patients in TDC—and you always wonder ‘how did you get this bad?’ Then you fix them and send them back and see them [again] two weeks later. It’s because they’re not getting a lot of the things you’ve recommended because a lot of it comes down to formulary issues. Being able to go over to the pill window twice a day, your medication is given 4 times a day—you’re missing out on two of those doses. There’s only certain types of things you can keep on yourself—KOP (Keep On Person) medications in the TDC. So it’s just sad, and it’s humbling—very humbling.”

Not all members of the care team take the time to develop such insight. As one third-year medical student explained:

“*Well we had a patient before who wasn't taking his medications and—the issue was the timing or something that he said—but nobody even wanted to look into it. [They’d say] ‘Well he's not going to take his medication, well fine. He can just go back and not be treated.’ But if it was a free-world patient they would never say that. They would go in and be like ‘why aren't you taking your medications?’ ‘What can we do to facilitate that?’ You know, it’s really different. And I think that varies a lot by the provider but there are people who feel that way who are just like ‘well you know…’ they just write them off. They don't give them the same chance.*”

Here, the moral judgment applied to incarcerated patients leads some members of the care team to be less vigilant, and indirectly contributes to the advanced pathology seen in those patients.

Some students also picked up on the enormous number of administrative hurdles contributing to the advanced pathology in incarcerated patients. “There’s also a lot of bureaucracy in prisons and a lot of times these diseases progress so far because of bureaucratic [red] tape,” one particularly astute first-year student observed. “You can’t see a physician in time, it takes 2 weeks, there’s a waiting list, there’s not a doctor on call, there’s not one available so they need to go off site and then that has to be authorized. As physicians if we could somehow cut down on the bureaucracy or somehow find a way to streamline certain people without them being terminally ill and dying today that would help a lot,” the student concluded sarcastically.

However, for most trainees the discovery of such advanced pathology was simply an opportunity to be cherished. “They have a lot more interesting diseases that you wouldn’t always see. You see stuff that you would never see anywhere else,” a third-year student remarked excitedly. “They have a lot of TB, and really advanced cancers that you don’t see very often. But I think it’s good for training.” Another first-year resident concurred: “I knew I’d be able to see a lot of things other institutions wouldn’t. Which is what I was excited about.”

For one first year medical student, the excitement started before the first footstep onto the campus. “I knew about the program because I actually drove next to a bus coming from Huntsville on my way to the interview. So I saw all these prisoners chained up waving at me while I’m driving so I knew when I got here. I was actually excited about it too—you see further progressions of disease because they receive less treatment,” he told us before catching himself, adding, “which is really sad.”

For another trainee, the advanced pathology of TDC patients was the deciding factor in his choice of residency program:

“*[I]t was one of the drawing factors, when I was interviewing here for UTMB I thought ‘Oh, I don't want to come to Galveston.’ I was going to use this place as a practice interview but then I came here, I loved the program, the opportunities with the TDC—and I'm interested in doing infectious disease so the TDC provides a lot of great pathology. Like all the fungal infections, stuff I wouldn't see at my medical school—I went to Texas A&M, Temple, Scott & White; didn't see much stuff. Maybe one or two HIV patients 3rd or 4th year. Here, my first work month was my first month of residency and I saw streptococcal meningitis, histoplasmosis, TB—very commonplace... it was kind of a drawing factor to TDC for me personally.*”

### Grateful obedience and easy compliance

For many incarcerated patients, the caring touch of a healer represents the only human kindness they’ve experienced in years. Combined with the desperation that must accompany a highly progressed disease state, the result is that many trainees experienced their incarcerated patients as kinder, more patient, and more thankful for their services than free-world patients. “I felt like a lot of them were nicer than the free-world patients because someone’s being nice to them. Which may be—at least from talking to them—something they’re not always used to,” a third-year student told us.

This also challenged prevailing stereotypes that medical trainees often had about people who are incarcerated. “They’re completely normal in the sense that they weren’t really hostile or aggressive,” another third-year student discovered. “They were open to healthcare; a lot of them, actually, are really grateful for receiving healthcare. Some of them had really great attitudes.” “They’re really grateful for receiving healthcare,” another third-year student repeated. “They’re not hostile towards me then I think, ‘well you might have done something bad but you’re not doing anything bad to me.’”

“I’ve had a couple that were extremely grateful and even smiling when I walk in,” another third-year student relayed. “I’m talking to them about how they’re feeling and sometimes they would have extended conversations with me, [saying] ‘yeah, y’all are doing the best y’all can,’ and ‘y’all work together really well’ and things like that. And I walked out of there feeling…feeling good!”

A third-year resident described in detail how her fears were completely upended by how nicely her incarcerated patients behaved. “They could be murderers or rapists, so I’m really scared because those people could be more aggressive,” she described. “But after entering the system, [I discovered that] they’re not different than the general population. Honestly, they are much nicer, they are less demanding compared to the general population so they are very appreciative of what you do for them.”

A second-year resident specifically linked the gratefulness of incarcerated patients to their past neglect: “For every bad patient there’s probably three—three or four—that are so thankful you’re there [agreement from group] because they’ve been trying to see someone for months or years. And then they’re just glad you see them and listen to them.”

This dynamic of gratefulness resulted in a number of additional perks for trainees. The first perk trainees spoke excitedly about was the easy compliance of incarcerated patients. “You get a lot of freedom,” one first-year student remarked. “The patient population is very receptive to your care... they feel appreciative.” “I feel like they’re more receptive to you [as a student],” a third-year medical student reported. “They’re actually happy to see you sometimes. They’re glad that they’re getting the attention and even just to have someone to talk to.” “[S]tudents are less likely to be kicked out of a room in TDC than they are at [the free-world hospital],” a first-year resident confirmed.

Another first-year resident discussed this compliance as a function of patient privilege: “I was at a private hospital and I feel like the patients there sometimes have a sense of privilege and there’s a little more demand for certain tests or certain specialists or they have certain requirements. I appreciate that the patients I’ve had [in the TDC] aren’t demanding, they take what the doctor says at face value and they’re very respectful and courteous and I think they appreciate the care they receive.”

“They’re usually happy to help,” 1 second-year resident chimed in. “If you say ‘Oh I just want to teach her about something’ and they’ll say ‘Oh, ok.’ They take off their gown [and say] ‘what you got? I’ll show you anything.’ And sometimes it’s easier to ask them than some rich bigwig from the county. They’re more down to earth.” Here, we see how cherished such easy compliance is at a teaching hospital, with a cheerful interpretation of the incarcerated patient’s motivation to assist that does not take into consideration the complexities of consent in a captive population.

### Freedom

A second perk was the freedom to practice procedures on incarcerated patients that learners would not be allowed to practice on free-world patients. “Let me tell you something,” a third-year resident admitted in a hushed tone, “I didn’t see it myself but some students told me in the [operating rooms] they would let them do more in TDC.” “It’s definitely true,” a third-year student confirmed. “I know people who’ve gotten to do things that you wouldn’t otherwise get to do.” “They know and they’re willing, nobody’s ever said no,” a second-year resident told us.

“When I was a medical student my resident let me put in central lines,” a second-year resident confided. “It’s probably more so in the surgical unit,” another resident attested. “I would also agree. The surgical services—when I was in medical school they absolutely let the medical students do a lot—in regards to helping with the surgical procedures or suturing or lines,” another second-year resident admitted. “When I was in surgery, like I was first assist on at least half the cases that I scrubbed into,” a third-year student guiltily added. Then, realizing how egregious her admission sounded, quickly clarified, “It was relatively simple things, y’know, like hernias and not anything crazy. But the attending was always there.”

One third-year medical student was particularly forthcoming in sharing the freedom he’d been granted. “[Y]ou’ll find that you get to do more as a learner—as a medical learner—in the TDC as opposed to the free-world because people in the free-world have opinions about who they want giving care. So y’know, if you’re on urology, like I was, free-world people don’t like getting rectal exams from students. TDC patients have no choice or they don’t care. And to me that was a big advantage—I mean, not that I’m really fond of rectal exams—but it was a good learning experience to be able to do these exams with no pressure of ‘if you mess this up, they’re going to complain to the hospital or complain to the attending’ or something like that. There’s no threat of um, y’know, retribution or whatever. Repercussions, yeah.”

Even some first-year medical students had already picked up on the cynicism behind the greater leeway trainees are given with incarcerated patients. “I don’t think that it’s ok because you’re not experienced and it’s like they’re your guinea pig and it’s a human life you’re dealing with… [and] if it goes wrong nobody cares anyways. That’s how I see it. Like, ‘I can get to practice and if I’m successful at what I’m doing, then fine. If it’s wrong, then who cares?’ That’s how I see it.”

“It’s good for our education, but I don’t know how patients feel about it—about being guinea pigs,” a third-year student reflected. “It’s sad because they’re almost used to having their opinions pushed aside and marginalized. Which I guess makes sense because they’re in prison. Most of them would just go with it because they assume they don’t get to make any decisions so they assume they don’t get to make medical decisions either. They don’t know they have the right to refuse treatment, I think.”

“You can even see [this attitude] in the faculty,” a second-year resident told us. “Attendings will often say, ‘Aw yeah, we can round on table rounds—I don’t really need to go over there.’ That happens. So I think we’d be lying if we said we didn’t think [students] probably do get to do more procedures and why. Because TDC [patients] are looked at as lesser people than our free-world [patients]... Maybe we shouldn’t, but in general, that’s probably how people see it.”

Here, patient gratefulness, easy compliance and an amenability to letting students practice on them combine to create a learning atmosphere that many trainees found ideal and extremely appealing. As one third-year resident explained:

“*You discover that it’s heaven there [group laughter]. There's a lot of social issues in the free-world that you don't get involved with in TDC. You spend less time talking to families and people trying to intervene in your management and direct you on what to do because usually the prisoners will agree to go the way you want. You would explain everything and get their informed consent but in the real world they would ask for a second opinion and argue with you and they have doctors from outside that try to jump in the picture and dictate what to do. These issues you don't find in the TDC setting*.”

### “No Questions Asked”

We probed participants to expand on these phenomena, and asked why they were permitted more freedom with incarcerated patients. “It’s all liability,” a third-year resident said flatly. A second agreed: “Yeah, I mean the possibility that anybody’s going to pursue this or if something wrong happened somebody’s going to go ahead and sue you or go after this—it’s very low in the TDCJ. In the free-world there is family [asking], ‘What happened? What went wrong? Who did it?’ They will ask a lot of questions. There’s no questions asked [in TDC].”

The impact of not having any family members to advocate on the patient’s behalf came up often, as something trainees really appreciated. “[Y]ou’re eliminating a lot of family social issues as far as who’s the medical power of attorney that’s making these decisions or ‘are you going home with this person or this person?’ That’s all kind of eliminated because they’re going back to their unit. We get a lot less of those complications and you can just concentrate on the medicine,” a second-year resident confirmed. A third-year medical student concurred: “[I]n the free-world getting a procedure done, they’ll say ‘Let me talk to my wife first’ or ‘Let me talk to my kids first’ whereas in TDC it’s more or less, ‘yeah, let’s go ahead and get that done.’ In some ways it’s slower and in some ways you can expedite the process.”

“[T]here’s no pressure and the other thing, there’s no medical/legal consequences,” another third-year resident admitted. A third-year student agreed: “I think it stems more from the lack of legal repercussions. It’s that you don’t have high-priced lawyers coming after you if you are in the TDC. Not that you are any less cautious or any less responsible in your medical actions. It’s just that you are a little less…your neck is a little less breathed down on in the TDC.”

“And also on the free-world side there is a length of stay issue,” a third-year resident informed us. “If the patient stays more than this amount of time then your length of stay is longer than other facilities. So they have a length of stay issue. That goes with quality measurements, so in the TDC they don’t have all that.

### Moral judgment

Even in treating free-world patients, medical trainees readily acknowledged that they were immersed in a culture—common to safety-net hospitals—where patients were morally judged often. “[A] lot of times—especially at UTMB—we judge the patients who don’t have that much money. We judge them because they don’t show up to their appointments; for example, at PCP [UTMB Primary Care Pavilion], people just don’t show up,” one third-year student commented. Treating incarcerated patients complicated this phenomenon.

First and foremost, trainees grappled with a kind of moral distress over the disparity between people who are incarcerated and who are able to receive healthcare compared to so many free-world persons denied care. “Sometimes they actually get better care than people who are not incarcerated,” one first-year resident lamented. “I have patients in the free world who, if they don’t have insurance or don’t have the funds, they don’t get what they need. But in TDC they usually will. In some ways they get better care.”

At times, the moral judgment of incarcerated patients manifested itself in less obvious ways, such as the degree to which a provider will go to make the patient comfortable. In discussing the quality of care provided to TDC patients, one first-year resident ranted, “I remember I had one [incarcerated] patient who said she was vegan and was requesting certain types of food. It was ridiculous,” she declared. “I actually told her, ‘you put yourself in this position where you’re in prison and now you’re in this hospital and if you wanted your life to be having vegan food and whole foods, you probably should have gone a different path.‘ So sometimes you have to tell the patient their requests are unreasonable, but that’s the only time I’ve really been annoyed. That’s when I felt, ‘Oh, they’ve done all these things to put themselves here and now they want all of this?!’”

Many students acknowledged that the quality of care provided to incarcerated patients was probably poorer, yet wondered whether incarcerated patients should be happy with whatever quality of medical care they receive, given the fact that they are prisoners. “It’s crazy the hurdles that you have to go through to get proper care or something resembling proper care in the TDC,” a third-year student remarked. “But on the other hand, it *is* free for the patient. So you get what you pay for. And you can’t feel too guilty given the fact that if they weren’t in prison they might not be getting anything. Y’know, they’re getting a free surgery, treatment, free medical care, staying in the hospital for free for two weeks. OK you can’t really complain too much.”

These feelings are exacerbated when the patient is on death row. “It just felt weird,” another participant commented. “[The patient] had terminal liver disease but…I don’t know. It felt like, ‘should we be investing all these resources into someone who is going to die soon?’ That’s how it felt—we’re investing all this money and all these resources, for what? You know you’re going to kill him; it made no sense.”

Seeing the results of the pervasive moral valences applied to TDC patients had a profound effect on one third-year medical student. “It teaches you not to judge your patients,” she explained. “Even in the free world we do that. And then when you see the extremes, the result of judging people, judging your patients. You see that on the extreme level as you do in TDC; people just straight out not getting care because you’re mad at them basically. You see how damaging that can be and so I think I’m less likely to judge patients in the free world because of it.”

However, many trainees expressed with certitude that the moral judgment of patients was not a factor in the TDC Hospital. “Usually the degree of whatever they do doesn’t affect us taking care of the patient,” one third-year resident attested. Another quickly concurred, asserting that “the way we practice, there is totally no difference. We treat them the same, I care about the TDC patient as I care about the free world patient. It doesn’t make any difference for us.”

Some of our participants observed that incarcerated patients are acutely aware that they are at risk of being morally judged by healthcare providers. As one third year medical student attested, incarcerated patients “don’t trust their doctors as much sometimes because they’re in prison and because they already have the perception they’re going to get worse care, which is true sometimes. So they’re a little more guarded than they would be if the same patient was in the free-world. So, I think it does affect the relationship. Because they assume you don’t like them already. As soon as you walk in the room they assume this…We had a lot of people who felt that way.”

Another third-year medical student confirmed, “We had one patient who was super meticulous—every time we gave him medications he’d write down their name, write down the medication and the dosage and he was always really skeptical of everything that the doctors would say. He would say ‘Would you do this the same way if I was free-world?’ I guess he felt like he was getting brushed aside.”

Incarcerated patients with terminal illnesses are often even more skeptical. “They’re not trusting, usually,” a third-year resident observed. “They won’t trust you if you tell them about doing the research and they’re like ‘Oh, because I’m a prisoner you’re going to do this to me.’ Even people who have advanced cancer in TDC; we have advanced cancer in the university hospital and we tell them, ‘there are no options at this point and it’s better for you to seek hospice care. Chemotherapy is only going to make you sicker and it’s going to kill you, you aren’t going to benefit.’ For the TDC patient we try to relieve their anxiety like ‘we’re not saying this because you’re a prisoner, we’re not trying to just get rid of you. It’s just that the disease is so advanced that we can’t do anything. You’re getting the same treatment as anybody outside.’ So this is a big anxiety for the patients.”

### Morbid curiosity

An additional factor complicating the moral judgment of incarcerated patients stemmed from the fact that, in Texas, as in many other states, the criminal records of incarcerated patients are publicly available online. Our data suggests an institutional culture at UTMB for persons involved in the care of incarcerated patients to research a patient’s criminal history, oftentimes before seeing the patient for the first time. This practice was often imparted to learners by more senior members of the medical care team—even attendings. As one MS-III admitted to us, “I had an attending on Endocrine who would Google it before we went into every room... He would Google them, every time we went into a room, to see what they did. I’m not sure why.”

Another third-year student confirmed, stating “Well yeah. He would look it up on his iPad and let us know. But he didn’t really judge them too much. He was just like, ‘Well, that’s interesting.’” There were no institutional rules or guidelines advising on such behavior, and many trainees were conflicted. As another third-year student relayed to us, “I remember I had on my inpatient team an intern [first year medical resident] and two upper level residents. The two upper-level residents were trying to figure out what our patient did [laughs] and the intern would just shut his ears and started humming.”

For many trainees, their curiosity just gets the better of them. One first-year student admitted, “I’ve been through a situation where I was definitely curious. It wasn’t even medically related, it was more just a curiosity thing. Seeing this person who’s polite and nice and here they are incarcerated so you’re curious. How, why, what was his life like, how did he get in that situation, how did he get here, what was the situation? So you just get curious, we’re nosy, we’re doctors.”

Many trainees expressed a similar curiosity, exacerbated when the patient was particularly nice or particularly rude. As a third-year student told us:

“*If your patient falls on either side of the spectrum--they're super nice and super grateful—you may ask, ‘what are you in for?’ or if they're extremely mean and extremely hostile then you might be like, ‘oh man, I bet he was in for something really bad.’ And then for those in between I guess people don't really care to look those up. I know certain people say they will look up everybody before; some people will only look up what their patients did after they've been discharged. Some people will absolutely outright refuse to know any of it.*”

One first-year medical student knew clearly why she would not want to look up a patient’s criminal record. “If I know what they did and it’s something that I felt strongly about, I may not even do it on purpose, but I may not do the hardest that I can. I may not do my best, not even on purpose. Just subconsciously.” Another first-year medical student, however, was convinced that knowing would actually make her a better doctor: “For me, knowing would make me a better physician because that’s how I talk to people and get to know them and treat them and have a conversation based on our dynamic. It’s going to confer bias, whether you know or you don’t know. The fact that you’re seeing an incarcerated patient will confer some sort of subconscious bias.”

### Training and orientation

Finally, we asked participants how well they felt the orientation prepared them for providing care to incarcerated patients. Surprisingly, UTMB provides no specialty training for dealing with any of these complex issues raised by trainees. There was security and procedural training, but no vulnerable populations or ethics instruction. As we discuss further in a subsequent publication, in the absence of such training, an institutional culture of correctional healthcare has prevailed where these exploitative power dynamics proliferate and replicate, in one generation of medical trainees after another.

## Discussion

Overall, we found that trainees cherish this liberated learning environment in a prison hospital, likely due to their own feelings of insecurity and vulnerability as learners, but many lack insight that their increased freedom is wholly dependent upon the non-freedom of the incarcerated patient. Learners’ desire to work with incarcerated patients comes from the removal of the sullying influence of social factors that mark free-world medicine. The distaste for the storied elements of human life is due to the belief that they get in the way of medical work, or as Foucault argues, dealing with the subjective elements of the illness experience interrupts the power of the “medical gaze,” that mode of perception that allows physicians to discern the hidden biological truths underpinning a patient’s signs and symptoms (Foucault, 2003). While pre-allopathic medicine relied on patients’ stories as the sole source of knowledge, the medical gaze prioritizes a scientific version of medical practice where pathology is ascertained through objective undisturbed observation of the body, thereby rendering a person’s self or identity irrelevant. With the advanced pathology of disease states in full bloom in the TDC hospital, medical trainees can properly focus on the prized technical aspects of their craft, unburdened by a patient’s story (as it is assumed false) and bypass having to face the distraction of seeing disease in the context and as a consequence of dehumanizing treatment.

Part of what trainees come to love about this environment is the easy compliance and hassle-free amenability of incarcerated patients—characteristics inextricably linked to the unique vulnerability of prisoners made possible through institutional neglect, isolation from family support, and lack of autonomy. In the above exchanges, we see that trainees appreciate an increased freedom to practice what they are learning, unencumbered by patient autonomy, annoying family interventions, and without fear of legal or professional repercussions if something goes wrong. The use of incarcerated patients for medical training exploits this vulnerability to the advantage of learners. The interactions between such patients and medical trainees are pregnant with dynamics of power/knowledge and discipline, in ways that remain largely obscured to the learners involved. Both incarcerated patients and medical trainees are at the bottom of rigid power hierarchies—one medicine, the other criminal justice—that leave both the patient and learner desperately dependent on one another: medical trainees seeking to hone the clinico-anatomical gaze and incarcerated patients in urgent need of a healer’s touch.

The production of vulnerability in the incarcerated patient begins with the suspicion of malingering. In all situations, healthcare providers make evaluative judgments of patients’ motives, the legitimacy of their symptoms, and the congruence between the physician’s and the patient’s conceptual model of illness (May et al., 2004). This we observed most clearly with the pervasive perception that incarcerated patients malinger. This context facilitates further exploitation and new kinds of vulnerability.

Corrections officers mediate all correctional healthcare, who have to agree or confirm that a person held as a prisoner seems ill before referring him or her to medical staff for further evaluation. However, correctional officers are taught to generally suspect malingering and manipulation as part of their training (Texas Department of Criminal Justice, 2017). For correctional officers in Texas, 3.25 out of a total 80 h of new officer training curriculum is dedicated to this topic (Miller, 2016). Scholarly estimates for the prevalence of malingering among the incarcerated range from 32 to 56% (McDermott & Sokolov, 2009).

Because of this widespread assumption, the resultant neglect of the illness complaints of incarcerated people can be severe or even fatal (Fathi, 2018; Mitchell, 2018). For incarcerated patients, this pervasive perception of malingering plays into a general reluctance among correctional officers to refer people who are incarcerated to outside medical care when they make health complaints, until irrefutable outward signs and symptoms are clearly observable. For incarcerated people in Texas, this manifests itself in a great deal of advanced pathology when incarcerated patients finally arrive at the TDC hospital.

As a concept, the idea of feigning illness dates back to antiquity (Charaka Club, 1941), but the first known use of the term “malingering” traces back to the early nineteenth century. From the French adjective *malingre* meaning “puny” or “sickly,” the English adoption of the word as a verb around the 1820s was most often used to describe the illness complaints of soldiers, sailors, and most notably, people who were held in bondage as slaves. Owners delayed and denied people held as slaves access to medical care by routinely accusing them of malingering (Hackford, 2004). Plantation healthcare was delivered as a form of social control, first and foremost simply in the acknowledgement of being legitimately ill. Physicians who treated people held as slaves were in a position of conflicted dual loyalty, torn between the needs of their patients and demands of the slave owners paying for their services (Boster, 2009). Their prognoses were a constant negotiation with slave owners to define what constituted a legitimate medical issue and, therefore, a valid excuse from work assignments. For instance, infectious diseases were recognized as medical illnesses while physical trauma, exhaustion, and rape were not (Stowe, 2004).

The forced institution of slavery was the major structural determinant of a slave’s health. Healthcare was administered not for the well-being of the slave but rather for the maintenance of the slave’s body to remain productive, and where the slave was the patient but not the client to whom the physician had primary fiduciary responsibility (Fett, 2002). So common was the perception of malingering that a student at the Medical College of South Carolina devoted his thesis to the feigned illnesses of persons held as slaves, with suggestions on the best whipping techniques to discern a malingering person held as a slave (McCloud, 1850). Other doctors suggested even more extreme methods, both physical and psychological, to detect malingering, such as threatening to pour boiling water on the subject’s legs but then using cold water instead, setting fire to the suspect’s clothes or bed, threatening castration, or most extremely, “the insertion of a red-hot ramrod into the rectum” (Keen, Weir, & Morehouse, 1864).

Certainly, the brutality of slavery provided motivation for people held as slaves to malinger, even as it extracted a genuinely severe physical and mental toll. Similarly, the brutality of imprisonment surely also provides motivation to malinger. In both situations, healthcare providers are tasked with being arbiters to discern legitimate illness, while accepting the imposition of the brutality from which the suspected malingering patient is seeking respite. From a Foucauldian analysis, we see that this preoccupation with malingering is critical practice for developing the medical gaze. Nevertheless, by acting as arbiters who alone could discern legitimate illness, physicians played a crucial role in the social control of persons held as slaves, keeping them healthy enough to endure the cruelties of the institution while certifying when they could and could not be worked. We find this dynamic unwittingly replicated in the provision of healthcare to the incarcerated.

The combination of lack of previous health insurance coverage, routine and preventive healthcare disincentives by the demanding of co-pays, the capitated managed care model that discourages patient encounters, and the pervasive perception of malingering all work together to produce an incarcerated body suffering from advanced pathologies, desperate for care, grateful for any services delivered, and amenable to almost any request made by medical learners. The excitement of medical trainees upon encountering the advanced pathology in incarcerated patients exposes their own insecurities about honing their skills at developing the clinical gaze and making sure that they do not ever miss a diagnosis. This illuminates one of the side effects of this new mode of medical perception in that the patient story in many ways becomes ultimately irrelevant to the empirical observations of the physician’s trained eye (Foucault, 2003). The ultimate truth that a person held as a prisoner might be malingering in order to stave off further trauma caused by imprisonment is ultimately dismissed as an issue not of the doctor’s concern.

As has been noted in numerous historical accounts of research involving prisoners, any request made of an incarcerated person by someone in a position of authority carries with it the risk of situational coercion, by the very nature of the prisoner’s state of complete non-freedom (Hornblum, 1998; Shuster, 1997; World Medical Assocation, 2013). One of the first and primary ways this vulnerability is instituted in incarcerated persons is that they lose the right to their own bodily integrity. They are made to strip naked whenever instructed by authorities, and made to submit to cavity searches. The medical student who, expressing with glee that incarcerated patients did not object to students practicing digital rectal exams on them because “TDC patients have no choice or they don’t care,” makes this assumption without fully comprehending that the strip-down body cavity search is one of the primary intake rituals initiating a person into their new, incarcerated status. In other words, one of the first things that happens to a person upon being imprisoned is the violation of their bodily integrity, and prisoners are violently reminded, if need be, that they do not have the right of refusal when it comes to this violation. This is not to mention the longstanding tradition of assaults and rapes perpetuated by corrections officers upon people incarcerated (Jacobs, 2004). By reproducing this violation of bodily integrity in the medical setting, even if only by the kinder, gentler probing of the inexperienced medical trainee, it reproduces the dynamic of powerlessness faced by incarcerated patients, thereby placing the delivery of healthcare squarely in the domain of disciplinary practices imposed on people who are incarcerated.

## Conclusions

It is this discipline that develops incarcerated patients into ideal medical subjects—easily compliant and amenable to allowing trainees to practice their skills on them. Medical trainees have their own insecurities and vulnerabilities: fear of being “pimped” by their superiors for not knowing enough, fear of being shunned by patients who do not want students involved in their care, fear of missing a diagnosis for being so inexperienced. These anxieties find relief in the freedom trainees are granted in the care of incarcerated patients. This freedom, however, is a form of power, founded on the very powerlessness of the incarcerated patient.

Without specific vulnerable-populations training, learners usually fail to recognize the easy compliance of incarcerated patients as desperation for help. The amenable disposition of the prisoner soothes the insecurities of the medical learner, and both parties develop a dependency on the other. In this manner, the delayed delivery of healthcare becomes a technique for controlling the incarcerated body in the medical setting, turning normally unruly bodies docile and amenable to being used as clinical training material. Most trainees did not make this connection on their own and remained blissfully oblivious, becoming part of an exploitative power dynamic without even realizing it.

Despite the few who had misgivings, a majority of the trainees we interviewed expressed that they were at no risk of letting moral judgments interfere with their professional obligation to deliver the same standard of care to incarcerated patients as they do free-world patients. The literature on the prevalence of moral judgments in the clinical encounter, however, suggests that this self-assuredness might be misplaced. Healthcare providers readily acknowledge struggling to maintain empathy in encounters with patients who trigger moral judgments (Cassell, 2004; Eisenberg, 1979; Justin, 2000; Kelly & May, 1982). The moral judgment of patients is pervasive, occurring not only with rude, bad-behaving or incarcerated patients but also in everyday situations in which appraisals of patients’ social worth and culpability are routine (Hill, 2010).

Historically, poor people have been at particular risk of moral judgment in American healthcare. Primary care physicians serving poor communities are often troubled by what they perceive as their patients’ lack of motivation to live healthy and their “dysfunctional behavioral characteristics” (Monnickendam, Monnickendam, Katz, & Katan, 2007; Willems, Swinnen, & De Maeseneer, 2005). What’s more, social outgroups are often concentrated at safety-net teaching hospitals: poor and homeless persons, those suffering from substance-use disorders, and the untreated mentally ill. These are the groups that most commonly trigger negative stereotypes and thus elicit both implicit and explicit bias from healthcare providers (Foster & Onyeukwu, 2003; Hill, 1992; Howard & Chung, 2000). This risk is all the more pertinent for incarcerated patients (Jones & Holmwood, 2005), whose identities intersect as both poor and, ostensibly, immoral. As one anthropologist noted in her ethnographic study of healthcare providers in an intensive care unit, the differences in both clinical care and comfort care given to regular patients versus those seen as morally culpable can be severe (Cassell, 2004).

In light of this evidence, we found the institutional culture—often imparted by attendings—of medical trainees looking up their incarcerated patients’ criminal records troubling in that it adds an additional risk of moral judgment to the clinical encounter that is likely ultimately harmful to the patient. This is also an expression of power exercised upon incarcerated patients by healthcare staff in that it is a further violation of their right to privacy. Free-world patients have the right to divulge as much or as little background information about themselves as they wish to their healthcare providers. Incarcerated people largely do not. While a few medical trainees recognized the danger, refused to look up patient criminal records and tried to avoid hearing the results when their peers did so anyway, the high percentage of students who believed that such knowledge posed no risks to the quality of care they deliver is troubling given the preponderance of previous research suggesting otherwise (Hill, 2010).

Researchers have also found that patients are acutely aware when they are socially situated so as to be at risk of being morally judged by healthcare providers. Such patients pay significantly more attention to impression management than do other patients (Malat, van Ryn, & Purcell, 2006). We suspect that impression management plays a large role in the friendly and amenable disposition of incarcerated patients as reported by our participants, compounding the vulnerability they face from neglect of their advanced pathological states of disease.

Not all prisoners are guilty of the crimes for which they have been convicted (Gross, Chen, Kennedy, & O'Brien, 2014; Poveda, 2001; Walsh, Hussemann, Flynn, & Golian, 2017). While this would be important for healthcare providers to consider upon treating incarcerated patients, focusing on the question of a patient’s individual guilt risks implying to providers that incarcerated persons whose guilt *is* certain actually *are* undeserving of the same standard of care. Instead, a correctional health curriculum may benefit from a broader consideration of the politics of crime and punishment in the United States. This would include an analysis of the large numbers of persons incarcerated for lack of adequate mental healthcare and untreated substance use disorders (Baillargeon et al., 2009). It should also include an examination of the racial and class-based discriminatory practices that ultimately lead to a person being incarcerated, such as: what communities are targeted and surveilled for criminal activity (Kirk, 2008; Warren & Tomaskovic-Devey, 2009); disparities in arrests made for similar crimes (Austin & Allen, 2000; Smith, Visher, & Davidson, 1984); disparities in the charges police file when they make an arrest (Crutchfield, Skinner, & Haggerty, 2012); and disparities in prosecutorial discretion, judicial sentencing and punishment (Spohn, 2014). Ultimately, the guilt or innocence of an incarcerated patient shouldn’t matter, and that is why healthcare providers should not research the criminal records of patients.

The medical trainees in this study were provided an orientation that only focused on security, safety, the prison health system, and logistics. The timing and frequency of the orientation was generally inconsistent, with residents receiving the training multiple times whereas medical students often received their orientations *after* beginning their rotation or sometimes, not at all. With the focus mainly on security, trainees noted that it reinforced the idea of incarcerated patients as an inherently violent and dangerous class of patients. It was only once they started rotations in the TDC hospital that they learned that this was not the case. While security protocols are important, they should be balanced with more information to help learners understand the immense precarity of incarcerated patients.

As AMCs take on the mantle of caring for the incarcerated, be it for motivations of guaranteed reimbursement, good clinical training material for medical learners, or for increased research opportunities, they will need to implement special training to teach healthcare providers how to adequately care for such a vulnerable population and how to avoid exploiting the imbalance of power between provider and patient. Without such training, however, this exploitative power dynamic risks being replicated in any additional academic medical settings where incarcerated patients are provided care.

### Limitations

Our qualitative approach and analysis is robust, but is also limited to the perspective of medical trainees. To develop a fuller picture of the medical care delivered to incarcerated patients, a similar study should be undertaken with nursing staff, corrections officers, attendings, hospital staff who are responsible for scheduling procedures and prioritizing laboratory work, and incarcerated patients. Also, we abstained from collecting more detailed demographic information about our subjects to help maintain confidentiality. This may obscure differences of opinion and perception that vary by race or ethnicity in our findings. Future research could also more precisely identify why prison populations have higher rates of advanced pathology, differentiating how much of it is due to late diagnoses and treatment quality vs premorbid conditions. Although the link between provider bias and disparate treatment is well established, this study does not measure inequities in health outcomes. It does, however, help illuminate the different points of contact where disparities likely take place. Findings presented here would greatly benefit from more systematic studies that demonstrate unequal care for the incarcerated that are alluded to by medical trainees.

## Data Availability

The datasets used and analyzed for this study are available from the corresponding author upon reasonable request.
